# Supplementary material for: Machine learning generalizability across healthcare settings: insights from multi-site COVID-19 screening
Source: NPJ Digit Med. 2022 Jun 7;5:69. doi: 10.1038/s41746-022-00614-9 (PMC9174159; doi:10.1038/s41746-022-00614-9)
Supplement: Supplementary file 1 — Supplementary Material [file 41746_2022_614_MOESM1_ESM.docx]

**Supplementary Information:**

**Machine Learning Generalizability Across Healthcare Settings: Insights from multi-site COVID-19 screening**

Jenny Yang, Andrew A. S. Soltan, David A. Clifton

**Supplementary Note 1:**

Inclusion & Exclusion Criteria:

*Oxford University Hospitals NHS Foundation Trust (OUH):* We included all patients attending acute and emergency care settings at OUH who received routine blood tests on arrival, considering presentations before December 1, 2019, and thus before the pandemic, as the COVID-19-negative (control) cohort. We considered presentations during the ‘first wave’ of the UK COVID-19 pandemic (December 1, 2019 to June 30, 2020) with PCR confirmed SARS-CoV-2 infection as the COVID-19-positive (cases) cohort. We excluded patients who opted out of electronic health record (EHR) research and those who did not receive laboratory blood tests or were younger than 18 years of age. Due to incomplete penetrance of testing during the first wave of the pandemic, and imperfect sensitivity of the PCR test, there is uncertainty in the viral status of patients presenting during the pandemic who were untested or tested negative. We therefore selected a pre-pandemic control cohort during training to ensure absence of disease in patients labelled as COVID-19-negative. Clinical features extracted for each presentation included first-performed blood tests, blood gases, vital signs measurements and PCR testing for SARS-CoV-2 (Abbott Architect [Abbott, Maidenhead, UK], TaqPath [Thermo Fisher Scientific, Massachusetts, USA] and Public Health England-designed RNA-dependent RNA polymerase assays).

*Portsmouth Hospitals NHS Foundation Trust (PUH):* PUH considered all patients admitted to the Queen Alexandria Hospital, serving a population of 675,000 and offering tertiary referral services to the surrounding region, between March 1, 2020 and February 28, 2021. Confirmatory COVID-19 testing was by laboratory SARS-CoV2 RT-PCR assay, considering any positive PCR result within 48hrs of admission as a true positive.

*University Hospitals Birmingham NHS Foundation Trust (UHB):* UHB considered all patients admitted to The Queen Elizabeth Hospital, Birmingham, between December 01, 2019 and October 29, 2020. The Queen Elizabeth Hospital is a large tertiary referral unit within the UHB group which provides healthcare services for a population of 2.2 million across the West Midlands. Confirmatory COVID-19 testing was performed by laboratory SARS-CoV-2 RT-PCR assay.

*Bedfordshire NHS Foundation Trust (BH):* BH considered all patients admitted to Bedford Hospital between January 1, 2021 and March 31, 2021. BH provides healthcare services for a population of around 620,000 in Bedfordshire. Confirmatory COVID-19 testing was performed on the day of admission by point-of-care PCR based nucleic acid testing [SAMBA-II & Panther Fusion System, Diagnostics in the Real World, UK, and Hologic, USA].

**Supplementary Note 2:**

Summary Statistics Across Different Sites:

The training set comprised 114,957 patient presentations to OUH prior to the global COVID-19 outbreak (November 30, 2017 - December 01, 2019), considered as COVID-19-free presentations, and 701 patient presentations during the first wave of the UK COVID-19 epidemic (December 01, 2019 - June 30, 2021) who had a positive PCR test for COVID-19. 72,223 patients were included across four validation cohorts, of whom 4,600 had a positive confirmatory test for COVID-19.

*Population Statistics:* In the external cohorts, patients admitted to PUH and BH trusts were similar ages (69 years (IQR 34) and 68 years (34); Kruskal-Wallis p=0.9448), however patients admitted to UHB were significantly younger (63 years (37); p<0.0001 & <0.0001). A higher proportion of patients admitted to UHB were female (53.1%) than PUH and BH (45.0% and 46.7%; chi-square p<0.000) and reported a South Asian ethnicity (13.2% versus 0.5% and 2.0%; chi-square p<0.0001). Prevalence of COVID-19 was higher in the Bedfordshire cohort, owing to the evaluation period matching the timeline of the second wave of the UK COVID-19 epidemic (11.1% versus 5.29% (PUH) and 4.27 (UHB); Fisher’s exact test p<0.0001 & <0.0001).

*Machine Learning Features Statistics:* Across all cohorts, most features had a significant difference in population median (Kruskal-Wallis p<0.05), except for systolic blood pressure, urea, and mean cell volume, which were similar (p > 0.1). A complete summary (median and interquartile ranges) of vital signs and blood tests for all patient cohorts are presented in Supplementary Tables S1 and S2, respectively.

P-values are calculated using the Kruskal-Wallis Test, which was implemented using the statistics package from the SciPy library.

**Supplementary Table 1:** Distribution of vital signs, reported as median and interquartile ranges, for each patient cohort.

|  | Oxford University Hospitals (pre-pandemic & wave 1 cases, to 30 June 2020) | | Oxford University Hospitals | Portsmouth Hospitals University NHS Trust | University Hospitals Birmingham NHS Foundation Trust | Bedfordshire Hospitals NHS Foundation Trust | Kruskal-Wallis,  p-value |
| --- | --- | --- | --- | --- | --- | --- | --- |
|  | Prepandemic cohort | COVID-19-cases cohort | October 1, 2020 – March 6, 2021 | March 1, 2020 - February 28, 2021 | December 01, 2019 - October 29, 2020 | January 1, 2021 - March 31, 2021 |  |
| Respiratory Rate (breath/min) | 18.0 (16.0-19.0) | 20.0 (18.0-24.0) | 18.0 (16.6-19.0) | 17.0 (16.0-19.0) | 18.0 (17.0-20.0) | 18.0 (16.0-20.0) | <0.0001 |
| Heart Rate (beats/min) | 82.0 (71.0-96.0) | 88.0 (75.0-101.0) | 84.0 (72.0-97.0) | 82.0 (71.0-95.0) | 86.0 (73.0-101.0) | 84.0 (73.0-97.0) | <0.0001 |
| Systolic Blood Pressure (mmHg) | 132.0 (118.0-150.0) | 131.0 (115.0-146.0) | 134.0 (119.0-152.0) | 128.0 (114.0-146.0) | 136.0 (119.0-155.0) | 131.0 (116.0-149.0) | 0.182 |
| Diastolic Blood Pressure (mmHg) | 74.0 (65.0-84.0) | 74.0 (64.0-84.0) | 75.0 (65.0-85.0) | 76.0 (67.0-84.0) | 77.0 (68.0-87.0) | 78.0 (68.0-88.0) | 0.028 |
| Tympanic Temperature (C) | 36.5 (36.1-36.9) | 36.9 (36.3-37.6) | 36.3 (36.0-36.7) | 36.3 (36.0-36.8) | 36.7 (36.4-37.2) | 36.5 (36.4-36.9) | <0.0001 |

**Supplementary Table 2:** Distribution of blood test features, reported as median and interquartile ranges, for each patient cohort.

|  | Oxford University Hospitals (pre-pandemic & wave 1 cases, to 30 June 2020) | | Oxford University Hospitals | Portsmouth Hospitals University NHS Trust | University Hospitals Birmingham NHS Foundation Trust | Bedfordshire Hospitals NHS Foundation Trust | Kruskal-Wallis,  p-value |
| --- | --- | --- | --- | --- | --- | --- | --- |
|  | Prepandemic cohort | COVID-19-cases cohort | October 1, 2020 – March 6, 2021 | March 1, 2020 - February 28, 2021 | December 1, 2019 - October 29, 2020 | January 1, 2021 - March 31, 2021 |  |
| HAEMOGLOBIN (g/L) | 130.0 (116.0-142.0) | 130.0 (114.0-144.0) | 129.0 (114.0-142.0) | 129.0 (114.0-143.0) | 127.0 (113.0-140.0) | 134.0 (119.0-146.0) | 0.016 |
| WHITE CELLS (10^9^ l^-1^) | 8.45 (6.46-11.18) | 6.98 (5.14-9.72) | 8.94 (6.7-12.06) | 8.6 (6.7-11.3) | 9.4 (7.1-12.6) | 9.2 (6.9-12.5) | <0.0001 |
| PLATELETS (10^9^ l^-1^) | 249.0 (199.0-307.0) | 215.0 (163.0-283.5) | 251.0 (198.0-314.0) | 251.0 (199.0-312.0) | 247.0 (196.0-311.0) | 246.0 (196.0-310.0) | 0.003 |
| MEAN CELL VOL (fl) | 89.6 (86.0-93.4) | 90.2 (86.6-94.2) | 90.2 (86.6-94.2) | 89.0 (84.9-93.0) | 89.9 (86.2-93.6) | 88.0 (85.0-92.0) | 0.210 |
| NEUTROPHILS (10^9^ l^-1^) | 5.72 (3.99-8.36) | 5.11 (3.48-7.49) | 6.44 (4.4-9.55) | 5.9 (4.2-8.6) | 6.9 (4.7-10.0) | 6.8 (4.7-9.73) | <0.0001 |
| HAEMATOCRIT | 0.39 (0.35-0.42) | 0.4 (0.35-0.44) | 0.39 (0.35-0.43) | 0.39 (0.34-0.42) | 0.38 (0.34-0.42) | 0.39 (0.35-0.43) | 0.002 |
| LYMPHOCYTES (10^9^ l^-1^) | 1.51 (1.0-2.13) | 0.96 (0.65-1.38) | 1.31 (0.85-1.89) | 1.5 (0.97-2.2) | 1.3 (0.9-1.9) | 1.27 (0.86-1.83) | <0.0001 |
| MONOCYTES (10^9^ l^-1^) | 0.64 (0.48-0.85) | 0.49 (0.35-0.74) | 0.66 (0.48-0.89) | 0.63 (0.48-0.85) | 0.7 (0.5-0.9) | 0.66 (0.48-0.92) | <0.0001 |
| EOSINOPHILS (10^9^ l^-1^) | 0.1 (0.04-0.2) | 0.01 (0.0-0.06) | 0.07 (0.02-0.16) | 0.1 (0.02-0.2) | 0.1 (0.0-0.2) | 0.06 (0.02-0.16) | <0.0001 |
| BASOPHILS (10^9^ l^-1^) | 0.04 (0.03-0.06) | 0.02 (0.01-0.03) | 0.04 (0.02-0.06) | 0.04 (0.02-0.06) | 0.1 (0.0-0.1) | 0.05 (0.03-0.07) | <0.0001 |
| SODIUM (mM) | 138.0 (136.0-140.0) | 136.0 (134.0-139.0) | 138.0 (135.0-140.0) | 138.0 (136.0-140.0) | 137.0 (134.0-139.0) | 138.0 (136.0-140.0) | <0.0001 |
| ALBUMIN (g/L) | 36.0 (32.0-39.0) | 32.0 (28.0-35.0) | 36.0 (31.0-39.0) | 36.0 (31.0-40.0) | 36.0 (32.0-40.0) | 35.0 (31.0-39.0) | 0.006 |
| ALKALINE PHOSPHATASE (IU/L) | 80.0 (64.0-105.0) | 82.0 (64.0-108.0) | 84.0 (66.0-112.0) | 84.0 (67.0-109.0) | 90.0 (71.0-119.0) | 94.0 (74.5-122.0) | <0.0001 |
| ALT (IU/L) | 18.0 (13.0-28.0) | 25.0 (17.0-41.0) | 20.0 (13.0-33.0) | 19.0 (13.0-30.0) | 19.0 (13.0-30.0) | 20.0 (13.0-31.0) | <0.0001 |
| UREA (mM) | 5.3 (4.0-7.4) | 5.9 (4.2-9.07) | 5.7 (4.2-8.3) | 5.2 (3.8-7.6) | 6.2 (4.5-9.0) | 5.8 (4.2-8.3) | 0.874 |
| BILIRUBIN (umol/L) | 9.0 (6.0-13.0) | 9.0 (7.0-13.25) | 9.0 (6.0-14.0) | 10.0 (7.0-16.0) | 10.0 (7.0-15.0) | 10.0 (7.0-14.0) | <0.0001 |
| CREATININE (umol/L) | 73.0 (60.0-93.0) | 79.0 (65.0-106.0) | 74.0 (60.0-97.0) | 74.0 (60.0-96.0) | 78.0 (62.0-105.0) | 80.5 (65.75-104.0) | <0.0001 |
| eGFR (ml/min) | 85.0 (63.0-150.0) | 78.0 (53.0-150.0) | 84.0 (58.0-150.0) | 83.0 (60.0-90.0) | 76.0 (52.0-90.0) | 76.0 (54.0-90.0) | 0.011 |
| POTASSIUM (mM) | 4.0 (3.7-4.3) | 4.0 (3.7-4.3) | 4.0 (3.8-4.4) | 4.2 (3.9-4.4) | 4.1 (3.8-4.4) | 4.3 (4.0-4.6) | <0.0001 |
| CRP (mg/L) | 8.6 (2.3-39.0) | 72.5 (23.8-143.6) | 15.8 (3.5-67.4) | 13.0 (3.0-71.0) | 12.0 (3.0-61.0) | 10.7 (2.8-48.78) | 0.003 |

**Supplementary Table 3:** Final hyperparameter values used in models.

| Model | Nodes | Dropout Rate | Learning Rate | Epochs | Batch Size |
| --- | --- | --- | --- | --- | --- |
| OUH | 10 | 0.3 | 0.1 | 30 | 10 |
| PUH | 20 | 0.3 | 0.01 | 30 | 10 |
| UHB | 10 | 0.5 | 0.01 | 30 | 10 |
| BH | 10 | 0.3 | 0.01 | 30 | 10 |
| Multi-site | 10 | 0.5 | 0.01 | 30 | 10 |

**Supplementary Table 4:** Performance results of different models. Results are reported alongside 95% confidence intervals (CIs) based on standard error. CIs for AUROC are calculated using Hanley and McNeil’s method.

| Validation Site | Model | Sensitivity | Specificity | PPV | NPV | F1 | AUROC |
| --- | --- | --- | --- | --- | --- | --- | --- |
| OUH | Site-specific | 0.762 (0.744-0.781) | 0.844 (0.839-0.849) | 0.320 (0.307-0.333) | 0.974 (0.971-0.976) | 0.451 | 0.878 (0.868-0.888) |
|  | Multi-site | 0.843 (0.828-0.859) | 0.664 (0.658-0.671) | 0.195 (0.187-0.203) | 0.978 (0.975-0.980) | 0.317 | 0.838 (0.827-0.849) |
|  | Ready-made (PUH) | 0.814 (0.797-0.831) | 0.790 (0.784-0.795) | 0.272 (0.261-0.283) | 0.978 (0.976-0.980) | 0.408 | 0.879 (0.869-0.889) |
|  | Ready-made (UHB) | 0.795 (0.777-0.812) | 0.792 (0.786-0.797) | 0.269 (0.258-0.280) | 0.976 (0.973-0.978) | 0.402 | 0.867 (0.857-0.877) |
|  | Ready-made (BH) | 0.663 (0.642-0.683) | 0.905 (0.901-0.909) | 0.403 (0.387-0.420) | 0.965 (0.963-0.968) | 0.502 | 0.853 (0.843-0.864) |
|  | Threshold Adjustment (PUH) | 0.804 (0.787-0.822) | 0.811 (0.806-0.816) | 0.291 (0.279-0.303) | 0.977 (0.975-0.979) | 0.427 | 0.879 (0.869-0.889) |
|  | Threshold Adjustment (UHB) | 0.795 (0.777-0.812) | 0.792 (0.786-0.797) | 0.269 (0.258-0.280) | 0.976 (0.973-0.978) | 0.402 | 0.867 (0.857-0.877) |
|  | Threshold Adjustment (BH) | 0.813 (0.796-0.830) | 0.724 (0.718-0.730) | 0.221 (0.212-0.231) | 0.976 (0.973-0.978) | 0.348 | 0.853 (0.843-0.864) |
|  | Transfer learning (PUH) | 0.813 (0.796-0.830) | 0.800 (0.795-0.806) | 0.282 (0.270-0.294) | 0.978 (0.976-0.980) | 0.419 | 0.885 (0.875-0.895) |
|  | Transfer learning (UHB) | 0.799 (0.781-0.816) | 0.785 (0.779-0.791) | 0.264 (0.253-0.275) | 0.976 (0.974-0.978) | 0.397 | 0.872 (0.862-0.882) |
|  | Transfer learning (BH) | 0.830 (0.814-0.846) | 0.708 (0.702-0.714) | 0.215 (0.206-0.225) | 0.977 (0.975-0.980) | 0.342 | 0.862 (0.851-0.872) |
| PUH | Site-specific | 0.781 (0.740-0.823) | 0.886 (0.879-0.893) | 0.268 (0.242-0.294) | 0.987 (0.984-0.990) | 0.399 | 0.902 (0.882-0.923) |
|  | Multi-site | 0.854 (0.819-0.889) | 0.690 (0.679-0.701) | 0.128 (0.115-0.141) | 0.989 (0.986-0.992) | 0.223 | 0.858 (0.834-0.882) |
|  | Ready-made (OUH) | 0.633 (0.585-0.681) | 0.903 (0.897-0.910) | 0.259 (0.231-0.287) | 0.979 (0.975-0.982) | 0.368 | 0.861 (0.837-0.885) |
|  | Ready-made (UHB) | 0.755 (0.712-0.798) | 0.857 (0.849-0.865) | 0.220 (0.198-0.243) | 0.985 (0.982-0.988) | 0.341 | 0.883 (0.861-0.905) |
|  | Ready-made (BH) | 0.628 (0.579-0.676) | 0.916 (0.910-0.923) | 0.286 (0.255-0.316) | 0.979 (0.975-0.982) | 0.393 | 0.862 (0.838-0.885) |
|  | Threshold Adjustment (OUH) | 0.797 (0.757-0.837) | 0.784 (0.774-0.793) | 0.164 (0.147-0.181) | 0.986 (0.983-0.989) | 0.272 | 0.861 (0.837-0.885) |
|  | Threshold Adjustment (UHB) | 0.792 (0.751-0.832) | 0.833 (0.825-0.842) | 0.202 (0.182-0.222) | 0.987 (0.984-0.990) | 0.322 | 0.883 (0.861-0.905) |
|  | Threshold Adjustment (BH) | 0.786 (0.745-0.827) | 0.771 (0.761-0.781) | 0.155 (0.139-0.171) | 0.985 (0.982-0.989) | 0.259 | 0.862 (0.838-0.885) |
|  | Transfer learning (OUH) | 0.805 (0.765-0.844) | 0.845 (0.837-0.854) | 0.217 (0.196-0.239) | 0.988 (0.985-0.991) | 0.342 | 0.890 (0.868-0.911) |
|  | Transfer learning (UHB) | 0.805 (0.765-0.844) | 0.845 (0.837-0.853) | 0.217 (0.196-0.238) | 0.988 (0.985-0.991) | 0.342 | 0.892 (0.871-0.914) |
|  | Transfer learning (BH) | 0.792 (0.751-0.832) | 0.839 (0.831-0.848) | 0.208 (0.188-0.229) | 0.987 (0.984-0.990) | 0.330 | 0.885 (0.863-0.907) |
| UHB | Site-specific | 0.835 (0.759-0.911) | 0.822 (0.805-0.839) | 0.178 (0.142-0.215) | 0.991 (0.986-0.995) | 0.294 | 0.893 (0.849-0.937) |
|  | Multi-site | 0.879 (0.812-0.946) | 0.643 (0.622-0.664) | 0.102 (0.081-0.123) | 0.991 (0.986-0.996) | 0.183 | 0.864 (0.815-0.912) |
|  | Ready-made (OUH) | 0.714 (0.621-0.807) | 0.854 (0.839-0.870) | 0.185 (0.144-0.225) | 0.985 (0.979-0.991) | 0.293 | 0.878 (0.832-0.924) |
|  | Ready-made (PUH) | 0.780 (0.695-0.865) | 0.847 (0.831-0.862) | 0.190 (0.151-0.230) | 0.988 (0.983-0.993) | 0.306 | 0.866 (0.818-0.914) |
|  | Ready-made (BH) | 0.637 (0.539-0.736) | 0.893 (0.879-0.906) | 0.216 (0.166-0.265) | 0.982 (0.975-0.988) | 0.322 | 0.835 (0.783-0.887) |
|  | Threshold Adjustment (OUH) | 0.901 (0.840-0.962) | 0.669 (0.648-0.690) | 0.112 (0.089-0.135) | 0.993 (0.989-0.998) | 0.199 | 0.878 (0.832-0.924) |
|  | Threshold Adjustment (PUH) | 0.846 (0.772-0.920) | 0.755 (0.736-0.774) | 0.137 (0.109-0.166) | 0.991 (0.986-0.996) | 0.237 | 0.866 (0.818-0.914) |
|  | Threshold Adjustment (BH) | 0.901 (0.840-0.962) | 0.563 (0.541-0.585) | 0.087 (0.069-0.105) | 0.992 (0.987-0.997) | 0.159 | 0.835 (0.783-0.887) |
|  | Transfer learning (OUH) | 0.868 (0.799-0.938) | 0.735 (0.715-0.754) | 0.131 (0.104-0.158) | 0.992 (0.987-0.996) | 0.228 | 0.885 (0.840-0.930) |
|  | Transfer learning (PUH) | 0.835 (0.759-0.911) | 0.782 (0.764-0.800) | 0.150 (0.119-0.182) | 0.990 (0.985-0.995) | 0.255 | 0.874 (0.827-0.921) |
|  | Transfer learning (BH) | 0.879 (0.812-0.946) | 0.641 (0.620-0.662) | 0.102 (0.081-0.123) | 0.991 (0.986-0.996) | 0.182 | 0.852 (0.802-0.901) |
| BH | Site-specific | 0.724 (0.561-0.887) | 0.923 (0.886-0.959) | 0.568 (0.408-0.727) | 0.960 (0.933-0.987) | 0.636 | 0.902 (0.826-0.978) |
|  | Multi-site | 0.966 (0.899-1.000) | 0.662 (0.597-0.726) | 0.286 (0.196-0.375) | 0.993 (0.979-1.000) | 0.441 | 0.889 (0.809-0.969) |
|  | Ready-made (OUH) | 0.724 (0.561-0.887) | 0.908 (0.869-0.948) | 0.525 (0.370-0.680) | 0.959 (0.931-0.987) | 0.609 | 0.880 (0.798-0.963) |
|  | Ready-made (PUH) | 0.966 (0.899-1.000) | 0.812 (0.758-0.865) | 0.418 (0.300-0.536) | 0.994 (0.983-1.000) | 0.583 | 0.940 (0.880-1.000) |
|  | Ready-made (UHB) | 0.897 (0.786-1.000) | 0.889 (0.846-0.932) | 0.531 (0.391-0.670) | 0.984 (0.966-1.000) | 0.667 | 0.924 (0.856-0.992) |
|  | Threshold Adjustment (OUH) | 0.828 (0.690-0.965) | 0.841 (0.791-0.890) | 0.421 (0.293-0.549) | 0.972 (0.948-0.996) | 0.558 | 0.880 (0.798-0.963) |
|  | Threshold Adjustment (PUH) | 0.862 (0.737-0.988) | 0.913 (0.875-0.951) | 0.581 (0.434-0.729) | 0.979 (0.959-0.999) | 0.694 | 0.940 (0.880-1.000) |
|  | Threshold Adjustment (UHB) | 0.897 (0.786-1.000) | 0.899 (0.857-0.940) | 0.553 (0.411-0.695) | 0.984 (0.966-1.000) | 0.684 | 0.924 (0.856-0.992) |
|  | Transfer learning (OUH) | 0.759 (0.603-0.914) | 0.942 (0.910-0.974) | 0.647 (0.486-0.808) | 0.965 (0.940-0.991) | 0.698 | 0.904 (0.829-0.979) |
|  | Transfer learning (PUH) | 0.897 (0.786-1.000) | 0.908 (0.869-0.948) | 0.578 (0.433-0.722) | 0.984 (0.967-1.000) | 0.703 | 0.944 (0.885-1.000) |
|  | Transfer learning (UHB) | 0.931 (0.839-1.000) | 0.913 (0.875-0.951) | 0.600 (0.457-0.743) | 0.990 (0.975-1.000) | 0.730 | 0.928 (0.862-0.994) |

**Supplementary Table 5:** Mean results across ready-made models. Results are reported alongside 95% confidence intervals (CIs) based on standard error. CIs for AUROC are calculated using Hanley and McNeil’s method.

| Validation Site | Model | Sensitivity | Specificity | PPV | NPV | AUROC |
| --- | --- | --- | --- | --- | --- | --- |
| OUH | Ready-made | 0.757 (0.642-0.831) | 0.829 (0.784-0.909) | 0.315 (0.258-0.42) | 0.973 (0.963-0.980) | 0.866 (0.843-0.889) |
|  | Threshold Adjustment | 0.804 (0.777-0.830) | 0.776 (0.718-0.816) | 0.260 (0.212-0.303) | 0.976 (0.973-0.979) | 0.866 (0.843-0.889) |
|  | Transfer learning | 0.814 (0.781-0.846) | 0.764 (0.702-0.806) | 0.254 (0.206-0.294) | 0.977 (0.974-0.980) | 0.873 (0.851-0.895) |
| PUH | Ready-made | 0.672 (0.579-0.798) | 0.892 (0.849-0.923) | 0.255 (0.198-0.316) | 0.981 (0.975-0.988) | 0.869 (0.837-0.905) |
|  | Threshold Adjustment | 0.792 (0.745-0.837) | 0.796 (0.761-0.842) | 0.174 (0.139-0.222) | 0.986 (0.982-0.990) | 0.869 (0.837-0.905) |
|  | Transfer learning | 0.801 (0.751-0.844) | 0.843 (0.831-0.854) | 0.214 (0.188-0.239) | 0.988 (0.984-0.991) | 0.889 (0.863-0.914) |
| UHB | Ready-made | 0.710 (0.539-0.865) | 0.865 (0.831-0.906) | 0.197 (0.144-0.265) | 0.985 (0.975-0.993) | 0.860 (0.783-0.924) |
|  | Threshold Adjustment | 0.883 (0.772-0.962) | 0.662 (0.541-0.774) | 0.112 (0.069-0.166) | 0.992 (0.986-0.998) | 0.860 (0.783-0.924) |
|  | Transfer learning | 0.861 (0.759-0.946) | 0.719 (0.620-0.800) | 0.128 (0.081-0.182) | 0.991 (0.985-0.996) | 0.870 (0.802-0.930) |
| BH | Ready-made | 0.862 (0.561-1.000) | 0.870 (0.758-0.948) | 0.491 (0.300-0.680) | 0.979 (0.931-1.000) | 0.915 (0.798-1.000) |
|  | Threshold Adjustment | 0.862 (0.690-1.000) | 0.884 (0.791-0.951) | 0.518 (0.293-0.729) | 0.978 (0.948-1.000) | 0.915 (0.798-1.000) |
|  | Transfer learning | 0.862 (0.603-1.000) | 0.921 (0.869-0.974) | 0.608 (0.433-0.808) | 0.980 (0.940-1.000) | 0.925 (0.829-1.000) |

**Supplementary Table 6**: Optimization thresholds used during model evaluation.

| Validation Site | Model | Optimization Threshold |
| --- | --- | --- |
| OUH | Site-specific: | 0.052 |
|  | Multi-site: | 0.042 |
|  | Ready-made (PUH): | 0.064 |
|  | Ready-made (UHB): | 0.046 |
|  | Ready-made (BH): | 0.200 |
|  | Threshold Adjustment (PUH): | 0.074 |
|  | Threshold Adjustment (UHB): | 0.046 |
|  | Threshold Adjustment (BH): | 0.060 |
|  | Transfer learning (PUH): | 0.052 |
|  | Transfer learning (UHB): | 0.054 |
|  | Transfer learning (BH): | 0.032 |
| PUH | Site-specific: | 0.064 |
|  | Multi-site: | 0.042 |
|  | Ready-made (OUH): | 0.052 |
|  | Ready-made (UHB): | 0.046 |
|  | Ready-made (BH): | 0.200 |
|  | Threshold Adjustment (OUH): | 0.024 |
|  | Threshold Adjustment (UHB): | 0.040 |
|  | Threshold Adjustment (BH): | 0.074 |
|  | Transfer learning (OUH): | 0.056 |
|  | Transfer learning (UHB): | 0.058 |
|  | Transfer learning (BH): | 0.056 |
| UHB | Site-specific: | 0.046 |
|  | Multi-site: | 0.042 |
|  | Ready-made (OUH): | 0.052 |
|  | Ready-made (PUH): | 0.064 |
|  | Ready-made (BH): | 0.200 |
|  | Threshold Adjustment (OUH): | 0.018 |
|  | Threshold Adjustment (PUH): | 0.034 |
|  | Threshold Adjustment (BH): | 0.022 |
|  | Transfer learning (OUH): | 0.030 |
|  | Transfer learning (PUH): | 0.034 |
|  | Transfer learning (BH): | 0.016 |
| BH | Site-specific: | 0.200 |
|  | Multi-site: | 0.042 |
|  | Ready-made (OUH): | 0.052 |
|  | Ready-made (PUH): | 0.064 |
|  | Ready-made (UHB): | 0.046 |
|  | Threshold Adjustment (OUH): | 0.030 |
|  | Threshold Adjustment (PUH): | 0.156 |
|  | Threshold Adjustment (UHB): | 0.050 |
|  | Transfer learning (OUH): | 0.082 |
|  | Transfer learning (PUH): | 0.134 |
|  | Transfer learning (UHB): | 0.066 |

**Supplementary Table 7**: Comparison of model outputs (raw probabilities) of ready-made models “as-is” to outputs of the transfer learning models, per training site. The raw probability output of the threshold adjusted models are the same as those from the ready-made models “as-is”, as threshold adjustment occurs after a model is already trained. P-values are calculated using the Wilcoxon Signed Rank Test, which was implemented using the statistics package from the SciPy library.

| Validation Site | Training Site | p-value |
| --- | --- | --- |
| OUH | PUH | <0.0001 |
|  | UHB | <0.0001 |
|  | BH | <0.0001 |
| PUH | OUH | <0.0001 |
|  | UHB | <0.0001 |
|  | BH | <0.0001 |
| UHB | OUH | <0.0001 |
|  | PUH | <0.0001 |
|  | BH | <0.0001 |
| BH | OUH | <0.0001 |
|  | PUH | <0.0001 |
|  | UHB | <0.0001 |
